# Consultation frequency and general practitioners’ and practices’ characteristics

**DOI:** 10.1186/s12875-023-01996-9

**Published:** 2023-02-04

**Authors:** Laura Baudier, Nicolas Senn, Pascal Wild, Christine Cohidon

**Affiliations:** 1 Center for Primary Care and Public Health (Unisanté), University of Lausanne, Lausanne, Switzerland; 2 PW Consulting, Laxou, France

**Keywords:** Consultation frequency, General practitioner, Practice characteristics, Patients characteristics

## Abstract

**Background:**

High workloads generated by a few patients who consult very frequently can become huge burdens for general practitioners (GPs). Patient-related factors have been repeatedly associated with frequent consultations, but there is evidence that GPs can also influence that frequency. We investigated how patients, GPs and their practices’ organisational characteristics were associated with consultation frequency.

**Methods:**

Data came from the SPAM Prev (Swiss Primary Health Care Active Monitoring, Prevention in primary care) national, cross-sectional survey conducted in 2015–16, including 167 GPs and 1105 patients. GPs completed an online questionnaire focused on practice organisation. Patients randomly recruited in general practices completed a questionnaire with fieldworkers. Factors predicting consultation frequency were investigated using multilevel Poisson regression models.

**Results:**

Negative associations with consultation frequency were found for females (Incidence Rate Ratio (IRR) 0.94, 95%CI [0.88–1.01]), less compliant patients (IRR 0.91, 95%CI [0.84–0.98]), high self-perceived health status (IRR 0.8, 95%CI [0.75–0.84]) and physical exercise (IRR 0.87, 95%CI [0.81–0.94]). Consultation frequencies were higher among patients with sleeping problems (IRR 1.08, 95%CI [0.96–1.23]), psychological distress (IRR 1.66, 95%CI [1.49–1.86]), chronic diseases (IRR 1.27, 95%CI [1.18–1.37]) and treatment with medication (IRR 1.24, 95%CI [1.12–1.37]). Positive associations with consultation frequency were found among GPs working longer hours (IRR 1.21, 95%CI [1.01–1.46]). Using shared medical records (IRR 0.79, 95%CI [0.67–0.92]) were negatively associated with consultation frequency.

**Conclusion:**

GPs’ practices’ characteristics, like patients’, are predictive of patients’ consultation frequency, but those associations’ underlying mechanisms require further qualitative investigation. These new findings could help optimise intervention strategies and reduce healthcare costs.

## Introduction

The heavy workloads generated by a few patients consulting very frequently can become a huge burden on general practitioners (GPs) [1, 2]. The behaviour of these *frequent attenders* (FAs) consumes more healthcare resources, boosts physicians’ workloads and increases costs, not only in primary care but also in specialist care [3].

Numerous studies in previous decades tried to characterise and detect FAs, but varying definitions of FAs influenced outcomes [4–6] and complicated comparative research. In addition, each patient’s unique needs and health issues mean that setting an ideal number of consultations is difficult. This also raises the issue of appropriate or excessive care utilization. For some patients, frequent use of care will be a response to real health needs, but for others, it will be an inadequate behaviour.

Factors such as being female [1, 2, 4, 7–12], older [1, 3, 4, 7–9, 11, 13, 14], having one or more physical illnesses [2, 4, 8, 10–13, 15] and having a psychiatric disorder [8, 11, 12] have been recurrently associated with frequent consultations. Ferrari et al. [2] even found that the odds of being an FA increased by 2.83 with each additional diagnosis. Factors like having too high or too low a body mass index (BMI) [3, 12–14], a lower level of education [2, 3, 12, 16], medically unexplained symptoms [3, 16, 17], and social [2, 4, 8, 12] and economic [2, 8, 13, 15] difficulties have also repeatedly shown associations. Conversely, protective factors against frequent consultation are a higher level of education [10, 12], higher income [10], employment [12] and exercise [12, 13].

The review by Kivela et al. [3] characterised FAs with four traits: frequent visits to GPs, feeling that symptoms (with or without a medical reason) are difficult to control, and lower self-perceived health status and quality of life. Many studies have reported associations with a lower subjective health status [8, 14, 16, 18, 19].

Few studies have investigated the doctor–patient relationship’s associations with frequent consultation. However, FAs have been shown to consult much more with some GPs than others [20], leading Neal et al. [20] to hypothesise that some GPs might perpetuate or even cause frequent attendance by, for example, being more empathetic, prescribing according to patients’ wishes, taking more time over consultations or inviting patients to come back. Indeed, studies [21, 22] reviewing interventions involving FAs showed that only Bellón et al.’s [23] “7 hypotheses + team” intervention with GPs reported significant reductions in FAs’ consultations.

The evidence that GPs themselves can influence their patients’ frequency of attendance thus raises the question of whether practice characteristics could also have an impact. Since these relationships have not yet been thoroughly researched, the present study aimed to develop new insights into associations between GPs’ characteristics, their practices’ organisational characteristics and frequent consultation, thus deepening existing knowledge on FAs. This could help develop better intervention strategies for reducing consultation rates, GPs’ workloads and overall healthcare costs.

## Methods

### Study design and population

Data originated from the Swiss Primary Health Care Active Monitoring, Prevention in Primary Care (SPAM Prev) study, a national, observational, cross-sectional survey conducted in 2015–16 to investigate prevention in family medicine by examining data from GPs and patients. This was itself part of the SPAM programme, initiated in 2010, and aimed at collecting and monitoring data on patterns in family medicine practices nationally [24]. A SPAM research network was created by inviting a random sample of GPs to participate, taken from professional lists stratified by each canton. The acceptance rate to participate was globally around 5%. The network was evaluated as nationally representative of GPs’ sex, age and rurality [24].

In 2015, this network consisted of 277 GPs, 167 of whom participated in the SPAM Prev study (60.2%). The participants did not differ overall from the initial GPs in terms of gender and age. A random sample of each physician’s patients was also included, with all study participants recruited directly in general practices when visiting their GPs. The inclusion criterion was being aged 16 years old or more. The goal was to enrol 10 patients per GP, but 1105 patients were included (a mean of about 7 patients per GP).

### Data

The Human Research Ethics Committee of the Canton of Vaud approved the study (N°74/15), and data collection took place between August 2015 and May 2016.

GPs completed an online questionnaire including questions on sociodemographics characteristics (as sex, year of birth, practice location, linguistic region, age of the practice) and practice organisation (function, hours of work per week, duration of average consultation, number of other doctors in the practice, number of patients, practice opening hours, medical records, use of IT for medicale files/appointment/test results, use of shared medical records, medical assistant for check-ups/vaccination/advice).

In the practices, fieldworkers first explained the study’s purpose and how patient involvement was free and anonymous. If patients consented, then fieldworkers administered the questionnaire. All participants were also offered a physical examination, including measurements of blood pressure, waist circumference, weight and height (to determine BMI), carried out by the fieldworkers in a standardised way.

The patient questionnaire contained standard sociodemographic questions (sex, age, native country, social situation, level of education, employment, monthly income), a measurement of self-perceived health and questions related to patients’ opinions or beliefs regarding health education, prevention, lifestyle and risk behaviours (smoking, alcohol, cannabis, physical activity, vaccinations, stress, screening). The final section covered diseases and medication, including physical and somatic diseases as well as compliance. Primary healthcare use was measured using the question, “Before this consultation, how many times had you seen your GP (or another GP) in the last 12 months?” This was the study’s key variable of interest. In addition, we selected for the present study, the other variables potentially related to the latter in both the patient questionnaire and the physician questionnaire.

Both questionnaires were initially written in French and evaluated locally for feedback and to ensure comprehension. Professional translators then translated them into German and Italian, the other main languages spoken in Switzerland.

### Statistical analysis

The statistical analyses were performed using STATA software (Stata14.1). Associations between the frequency of consultations at GPs’ practices during the last year and other variables were examined one by one using multilevel Poisson regression models, including the GP as a random effect and all the other variables as fixed effects. All the variables associated with consultation frequency with a *p*-value ≤ 0.2 were selected to generate two final multivariate models (using manual stepwise selection). One final multivariate model (M1) was applied to include all the patients, whereas a second final multivariate model (M2) only included patients who had consulted their GP fewer than 45 times in the last year. This second model was generated to exclude patients whose excessive numbers of visits might unduly influence the results. Finally, we conducted sensitivity analysis as well, using a dichotomous variable to identify FA. We used the threshold of 12 visits the past 12 months according to the literature review by Kivela [3]. We then conducted a multilevel logistic regression (M3) with the same independent variables as in the M1 model.

## Results

Analyses were based on 1105 patients and 152 GPs, as for some GPs, patient’s data were missing. The patient sample included 625 (56.6%) women, had a mean age of 58 years old and was mainly (75.3%) born in Switzerland. Most patients had at least one chronic disease (54.7%) and were under treatment with medication (73.7%). The mean frequency of visits per year was 5.62 (Table 1).

**Table 1:** Description of patient characteristics (frequencies, % or mean) and number of visits according to these characteristics (mean and quartile 25 and 75, Q_25_-Q_75_)

	***N***	**Frequency (%) or mean (std)**	**Nb visits (mean)**	***Q***_***25***_***-Q***_75_*
**Patient characteristics**	1105			
**Sex**				
Male	479	43.39	5.78	2–8
Female	625	56.61	5.50	2–7
**Age**				
mean (s.d.)	1104	58.05 (18.56)	-	
**Country of birth**				
Switzerland	832	75.29	5.74	2–8
Other	273	24.71	5.24	2–7
**Marital Status**				
In a relationship, with child	232	21.03	4.95	2–6
In a relationship, without child	456	41.34	5.69	2–8
Single, with child	46	4.17	5.51	2–8
Single, without child	307	27.83	6.26	2–10
Living with parents	62	5.62	4.59	1–5
**Educational level**				
Obligatory schooling only	191	18.1	6.45	2–10
Upper-secondary	568	53.84	5.96	2–8
Tertiary education	296	28.06	4.36	1–5
**Employment status**				
Employed	425	40.36	4.81	2–6
Retired	466	44.25	5.96	2–10
Student	41	3.89	3.82	1–5
Unemployed	121	11.49	7.82	2–10
**Self-perceived health status**				
(0 to 100), mean (s.d.)	1100	70.64 (24.29)	-	
**Psychological distress (PHQ4)**				
Normal (or none)	760	73.22	5.20	2–6
Mild	198	19.08	6.05	2–8
Moderate to severe	80	7.71	8.94	3–12
**Chronic disease**				
No	483	45.35	4.64	1–6
Yes	582	54.65	6.43	2–10
**Treatment with medication**				
No	282	26.26	3.52	1–4
Yes	792	73.74	6.37	2–10
**BMI**				
Normal range (18.5–24.99)	422	43.19	5.12	2–6
Underweight (< 18.5)	25	2.56	7.38	2–10.5
Overweight and obese (> 25)	530	54.25	6.13	2–9
Total patient sample	1068		5.62	2–7

^*Quartile 25 and Quartile 75^

GPs were mostly men (69.1%), and they mostly practiced in urban areas (71.7%). Most practices were group practices (58.4%) and used information technology to manage medical files (71.7%). The mean consultation duration was about 20 min (Table 2).

**Table 2:** Description of general practitioners’ and practice characteristics (frequencies, % or mean) and number of visits according to these characteristics (mean and quartile 25 and 75, Q_25_-Q_75_)

	***N***	**Frequency (%) or mean (s.d.)**	**Nb visits (mean)**	***Q***_***25***_***-Q***_***75***_
**GP and practice characteristics**	152			
**GP Sex**				
Male	105	69.08	5.71	3.81–7.12
Female	47	30.92	4.89	3.44–5.83
**GP age**				
< 47 years	39	25.66	5.28	3.80–6.87
47–53 years	34	22.37	5.07	3.43–6.57
54–60 years	41	26.97	5.65	3.71–6.4
61–80 years	38	25	5.76	3.6–7.12
**Area**				
Urban	109	71.71	5.64	3.5–6.875
Rural	43	28.29	4.99	3.81–6.00
**Linguistic region**				
German	83	54.61	5.83	3.78–7.14
French	56	36.84	4.61	3.41–5.83
Italian	13	8.55	6.74	4.92–7.89
**Age of the practice**				
(years in operation), mean (s.d.)	149	17.65 (10.60)	-	
**Type of practice**				
Solo	39	26.17	5.64	4.14–7.11
Group, with other GPs	87	58.39	5.37	3.44–6.5
Group, with other GPs and specialists	23	15.44	5.69	4.00–7.12
**Number of patients**				
mean (s.d.)	79	2120.8 (1295.95)	-	
**Consultation duration**				
(minutes), mean (s.d.)	147	19.39 (5.35)	-	
**Number of interactions per half-day**				
Face to face, mean (std)	146	13.29 (5.26)	-	
On the phone, mean (std)	145	3.70 (3.14)	-	
Per e-mail, mean (std)	142	1.23 (1.23)	-	
**Hours worked per week**				
< 40 h	46	32.17	4.83	3.43–5.78
40–49 h	39	27.27	5.58	3.60–7.11
≥ 50 h or more	58	40.56	5.83	4.00–7.00
**Practice opening hours**				
≤ 8 h/day	75	54.74	5.72	4.12–7.11
> 8 h/day	62	45.26	5.03	3.33–6.5
**Use IT for medical files**				
No	41	28.28	5.73	4.25–6.57
Yes	104	71.72	5.27	3.50–6.58
**Shared medical records**				
No	53	36.55	6.25	4.60–7.14
Yes, only within the practice	76	52.41	4.74	3.27–5.90
Yes, also with external healthcare professionals	16	11.03	5.73	4.03–7.17
**Patients can ask questions by email**				
No	30	20.41	5.21	3.50–6.57
Yes	117	79.59	5.52	3.71–6.67
**Medical assistant suggests check-ups, vaccination or advice**				
No	35	24.82	5.53	4.25–6.8
Yes	106	75.18	5.43	3.50–6.57

In univariate analyses, consultation frequency decreased with female sex (Incidence Rate Ratio (IRR) 0.95, 95%CI [0.90–1.01]), less compliant patients (defined as regular non-compliance with medical prescriptions) (IRR 0.81, 95%CI [0.76–0.86]), high self-perceived health status (IRR 0.67, 95%CI [0.64–0.70]), physical exercise (IRR 0.77, 95%CI [0.73–0.82]) and higher educational levels—(IRR 0.93, 95%CI [0.86–1.00] for an upper secondary education and (IRR 0.7, 95%CI [0.64–0.77] for tertiary level education). In contrast, consultation frequency increased with age (IRR 1.17, 95%CI [1.12–1.22]), sleeping problems (IRR 1.22, 95%CI [1.10–1.34]), moderate to severe psychological distress (IRR 1.72, 95%CI [1.57–1.88]), chronic disease (IRR 1.39, 95%CI [1.32–1.48]) and being treated with medication (IRR 1.71, 95%CI [1.59–1.84]). Regarding practice characteristics, consultation frequencies were higher among GPs who worked ≥ 50 h per week (IRR 1.21, 95%CI [1.02–1.43]) and varied according to the linguistic area. Longer opening hours and shared medical records were negatively associated with consultation frequency (IRR 0.86, 95%CI [0.74–0.99] and IRR 0.75, 95%CI [0.64–0.87], respectively) (Table 3).

**Table 3:** Factors associated with the frequency of consultations with GPs using Poisson and logistic regressions

	***N***	**Univariate**		**M1**^1^		**M2**^2^		**M3**^**3**^	
		**IRR**	**95% CI**	**IRR**	**95% CI**	**IRR**	**95% CI**	**OR**	**95% CI**
**Patients’ characteristics**	1105								
**Sex**									
Male	479	1		1		1		1	
Female	625	0.95	0.90–1.01	0.94	0.88–1.01	0.89	0.83–0.96	0.90	0.57–1.42
**Age**	1104	1.17	1.12–1.22	0.87	0.82–0.93	0.87	0.82–0.93	0.88	0.58–1.35
**Educational level**									
Obligatory schooling only	191	1		1		1		1	
Upper-secondary	568	0.93	0.86–1.00	0.94	0.86–1.03	1.06	0.97–1.16	0.98	0.57–1.71
Tertiary level	296	0.7	0.64–0.77	0.81	0.73–0.90	0.81	0.72–0.90	0.78	0.39–1.58
**Self-perceived health status**									
(0 to 100)	1100	0.67	0.64–0.70	0.8	0.75–0.84	0.78	0.73–0.82	0.62	0.45–0.85
**Physical exercise**									
No	300	1		1		1		1	
Yes	797	0.77	0.73–0.82	0.87	0.81–0.94	0.91	0.85–0.99	0.93	0.56–1.55
**Psychological distress**									
Normal	760	1		1		1		1	
Mild	198	1.11	1.04–1.20	1.11	1.02–1.21	1.13	1.04–1.24	1.41	0.81–2.48
Moderate to severe	80	1.72	1.57–1.88	1.66	1.49–1.86	1.41	1.25–1.59	2.68	1.29–5.57
**Sleeping problems**									
No	1001	1		1		1		1	
Yes	75	1.22	1.10–1.34	1.08	0.96–1.23	1.17	1.04–1.33	0.70	0.29–1.68
**Chronic disease**									
No	483	1		1		1		1	
Yes	582	1.39	1.32–1.48	1.27	1.18–1.37	1.19	1.10–1.28	1.66	0.98–2.80
**Treatment with medication**									
No	282	1		1		1		1	
Yes	792	1.71	1.59–1.84	1.24	1.12–1.37	1.31	1.18–1.46	1.42	0.65–3.10
**Taking anticoagulants**									
No	905	1		1		1		1	
Yes	197	1.65	1.54–1.75	1.58	1.45–1.71	1.64	1.51–1.78	3.64	2.18–6.06
**Compliant**									
Yes always	732	1		1		1		1	
Other	308	0.81	0.76–0.86	0.91	0.84–0.98	0.94	0.86–1.01	0.78	0.46–1.34
**BMI**									
Normal range (18.5–24.99)	422	1		1		1		1	
Underweight (< 18.5)	25	1.35	1.14–1.59	1.26	1.03–1.53	0.9	0.71–1.14	2.34	0.65–8.48
Overweight and obese (> 25)	530	1.14	1.07–1.21	0.99	0.92–1.06	1.0	0.93–1.07	1.21	1.29–5.57
**GP and practice characteristics**	152								
**Linguistic region**									
French	56	1		1		1		1	
German	83	1.25	1.07–1.45	1.14	0.97–1.35	1.10	0.94–1.28	1.81	1.00–3.29
Italian	13	1.5	1.16–1.95	1.42	1.06–1.90	1.41	1.09–1.83	3.15	1.42–6.97
**Hours worked per week**									
< 40 h	46	1		1		1		1	
40–49 h	39	1.14	0.94–1.37	1.15	0.95–1.40	1.18	0.99–1.40	1.62	0.86–3.05
≥ 50 h	58	1.21	1.02–1.43	1.21	1.01–1.46	1.25	1.06–1.48	2.03	1.13–3.67
**Practice opening hours**									
≤ 8 h/day	75	1		1		1		1	
> 8 h/day	62	0.86	0.74–0.99	0.88	0.76–1.02	0.85	0.75–0.98	0.97	0.92–1.02
**Shared medical records**									
No	53	1		1		1		1	
Yes, only within the practice	76	0.75	0.64–0.87	0.79	0.67–0.92	0.8	0.69–0.93	0.55	0.34–0.90
Yes, also with external healthcare professionals	16	0.9	0.71–1.15	1.02	0.79–1.31	1	0.80–1.26	0.98	0.51–1.89
Practice variance				0.131		0.099		1.09 10^–33^	

This table only displays variables that were significant in the multivariate models

CI = confidence interval; IRR = incidence rate ratio

^1^M1 = final multivariate model 1, including all patients

^2^M2 = final multivariate model 2, only includes patients with fewer than 45 visits per year

^3^M3 = Multivariate logistic model using a threshold of 12 visits in the past 12 months to define frequent users (estimated parameter is odds ratio, OR)

All the results outlined here concern the M1 multivariate analysis; results for M2 are in Table 3. Negative associations with consultation frequency persisted for female sex (IRR 0.94, 95%CI [0.88–1.01]), high self-perceived health status (IRR 0.8, 95%CI [0.75–0.84]), physical exercise (IRR 0.87, 95%CI [0.81–0.94]) and for less compliant patients (IRR 0.91, 95%CI [0.84–0.98]). Concerning education, the decrease in the number of visits persisted in both models for tertiary education (IRR 0.81, 95%CI [0.73–0.90]), whereas for an upper secondary education, it attenuated in M1 (IRR 0.94, 95%CI [0.86–1.03]) and totally disappeared in M2. Both final models associated more frequent consultations with moderate to severe psychological distress (IRR 1.66, 95%CI [1.49–1.86]), taking anticoagulants (IRR 1.58, 95%CI [1.45–1.71]) and, although these were attenuated, being under treatment with medication (IRR 1.24, 95%CI [1.12–1.37]) and having a chronic disease (IRR 1.27, 95%CI [1.18–1.37]) or sleeping problems (IRR 1.08, 95%CI [0.96–1.23]). All associations with BMI disappeared except for the positive association between the frequency of consultations for underweight people, which persisted, although attenuated, with an IRR of 1.26 (95%CI [1.03–1.53]). Negative associations with age were observed in both final models. No other patient attributes were significantly associated with consultation frequency in the multivariate analyses. Regarding practice characteristics, associations between consultation frequency and linguistic regions disappeared in both final multivariate analysis models. Both models still associated less frequent consultations with practices that shared medical records (IRR 0.79, 95%CI [0.67–0.92]) (Table 3).

Finally, in the M3 model, all associations with patients’ sociodemographic characteristics (gender, age, education level) disappeared. In contrast, associations with health-related variables remained positive, as well as the association with GP’s long working hours. Using a shared medical record with the practice remained associated with less FA (Table 3).

## Discussion

### Summary of main findings

A high self-perceived health status, physical exercise, less compliance, a higher educational level and female sex were the patient-related factors associated with lower use of primary care in this study. On the contrary, sleeping problems, moderate to severe psychological distress, chronic disease and treatment involving medication, particularly anticoagulants, were associated with higher use of primary care. As for practice characteristics, shared medical records were negatively associated with consultation frequency, whereas long GP working hours (≥ 50 h) per week was positively associated with consultation frequency.

### Comparison with existing literature

Most of our results concerning patients’ characteristics were supported by previous findings. Associations between frequent consultations and lower self-perceived health are well established [15, 17, 19, 25], with self-perceived health status being mentioned as the most significant factor influencing consultations with GPs [26]. Likewise, physical exercise was previously found to be a predictive factor of fewer consultations [12, 13], suggesting that people who exercise are usually in better health and less susceptible to diseases. However, our result persisted after adjusting for chronic disease, psychological distress and treatment with medication, which means that physical exercise may be an independent factor.

Our results supported previous works finding positive associations between consultation frequency and chronic disease [1, 8, 9, 14, 16, 27, 28], moderate to severe psychological distress (PHQ4) [5, 16] and treatment with medication [4, 10]. Physical and mental diseases, like psychological distress, can require regular follow-up and may exacerbate or progress into other diseases, thus explaining higher consultation rates. Besides, treatment with medication often requires monitoring and adjustment but can also cause side effects that lead to more consultations.

Our association between FAs and lower educational levels has also been demonstrated multiple times [2, 3, 13, 17], and higher educational levels have been shown to be a protective factor [10, 12]. We hypothesised that people with a higher level of education might use the gatekeeper system provided by primary care less often and go directly to specialists.

Results in the literature either established that females consulted physicians more frequently than males [1, 2, 7–12, 16] or that there was no significant association between sex and FAs [15]. In contrast, we found that female sex was associated with fewer consultations with GPs. Some studies [10, 12] have postulated that women consulted more frequently due to gynaecological problems. Indeed, in Jorgensen’s study [12], the effect on consultation frequency attenuated the most when female reproductive factors (parity, use of post-menopausal hormone replacement therapy, and contraceptives) were included in the model. One Swiss report [26] showed that women living in areas with fewer gynaecologists do not compensate by consulting gynaecologists in other areas, and it hypothesised that they consulted GPs instead. This could explain the difference between our results and those from other countries where there may be less access to gynaecologists. Another explanation may be that our population was directly selected at GPs’ practices.

The positive associations between health-related variables and FA were observed regardless the model (M1, M2, M3). In particular, in the M3 model, these associations were strong, in contrast with associations with patients’ sociodemographic characteristics that were no longer observed. These results tend to support the hypothesis of appropriate use of PC system by FA. For less frequent users, the use of care seems to be related to both healthcare needs and sociodemographic behaviours.

Less compliant individuals consulted their GPs less frequently, which could be interpreted in different ways: either non-compliant patients avoid consultations because their GP would probably urge them to take their medication and return for follow up or, on the contrary, patients who consult their GP less frequently are less compliant because they do not get enough advice and follow up. Therapeutic education may play a role and influence consultation frequency among these patients.

We found a significant positive association between consultation frequency and age in the univariate analysis; however, this association was reversed in our multivariate models. This is due to the inclusion of other independent variables correlated with age, such as the existence of chronic diseases and long-term treatments, which are positively associated with FA. The “positive effect” of age is therefore confounded with (and thus, entirely explained by) these variables in the univariate analysis. However the reversal of sign in the effect of age when adjusting on the confounders, should not be overinterpreted, as the population without any of the included health outcomes includes few elderly patients.

In agreement with previous findings in the literature [12–14], however, we did find positive associations between consultation frequency and underweight (BMI < 18.5) and overweight patients (BMI > 25). However, only the association with underweight patients persisted in our multivariate model. This could be explained by confounding factors: diseases associated with obesity (diabetes, hypertension) can require follow-up by a GP, thus reducing the independent effect of obesity [29]. Moreover, there is a significant social gradient regarding obesity [30]: people with an educational level corresponding to obligatory schooling only are at a greater risk of obesity than those with a university education. This could also be a confounding factor.

Some practice characteristics were also associated with consultation frequencies. Sharing medical records was associated with fewer consultations, which may be explained by better organisation, better continuity of care and greater efficiency. We could find no precedents for such an association in the literature, and this result could be a strong argument in favour of promoting the widespread introduction of electronic health records. Finally, the more hours a GP worked (⩾ 50 h) per week was associated with more frequent consultations. It is not easy to interpret in which direction this association might go, however. Indeed, if consultations are more frequent, workloads increase as well, which results in more hours worked per week.

### Study strengths and limitations

The present study’s main strength was its large sample size and the inclusion of patients’, GPs’ and practices’ characteristics. We were able to study associations between practice characteristics and patients’ consultation frequencies. To the best of our knowledge, this was unprecedented. Another strength of this study was using the frequency of consultations as a continuous variable. This enabled us to determine which factors were associated with an increase or a decrease in frequency without having to use a threshold or define *frequent attendance*.

The study nevertheless had some limitations. Social desirability bias is generally present in self-reported responses [27], and most of our information was from a self-reported questionnaire, not from observed data. Any errors caused by this were likely to be small; however, computer-based data from medical files would probably have been more reliable.

All the study participants were recruited directly in general practices, so patients who rarely or never visit a GP (or who have no time to fill the questionnaire) were under-represented. Another limitation is that we could only adjusted the analyses on the mean duration of consultation by GP. It was not possible taking into account the duration of each consultation. Consultation length might influence consultations frequencies.

Finally, the cross-sectional design implied that one must be careful when interpreting the associations causally.

## Conclusion

It seems that patients’ individual characteristics remain the most predictive factors of consultation frequency in primary care, especially those related to their physical and mental health. This implies that visits to a GP are usually related to a health issue and are therefore relevant. It is important to emphasise that there is no ideal number of consultations per year with a GP — everything depends on the patient’s health status and needs.

However, as mental health issues are one of the most predictive characteristics for more consultations with a GP, some patients might not be in the right place and would benefit from specialist care, which would help diminish the frequency of consultations and GPs’ workloads. Integrating psychologists into group practices might help solve this problem.

Some GP-related factors, for example, the influence of the duration of consultations, were not explained in this paper and deserve further analysis. Concerning practice characteristics, practice organization may be a very pertinent topic as we showed that using shared medical records was associated with fewer consultations. Better organisation, more efficiency and enhanced continuity of patient care could reduce the frequency of consultations, and this is something any healthcare system should aim for. More research on this subject is necessary, and new organisational models should be discussed and evaluated, such as the potential role of advanced practice nurses and which tasks they could take on to help reduce GPs’ workloads.

## Data Availability

Data could be available from the corresponding author on reasonable request.

## Abbreviations

GP: General practitioner
FA: Frequent attender
BMI: Body mass index
